# The Sigma‐2 Receptor/TMEM97 Agonist PB28 Suppresses Cell Proliferation and Invasion by Regulating the PI3K‐AKT‐mTOR Signalling Pathway in Renal Cancer

**DOI:** 10.1111/jcmm.17047

**Published:** 2021-11-16

**Authors:** Bo Zhan, Zhe Zhang, Chiyuan Piao, Xiao Dong, Yang Du, Chuize Kong, Yuanjun Jiang

**Affiliations:** ^1^ Department of Urology The First Hospital of China Medical University Shenyang People’s Republic of China

**Keywords:** Drug Resistance, EMT, PB28, PI3K‐AKT‐mTOR pathway, Renal cancer, Sigma‐2/TMEM97

## Abstract

Sigma‐2 receptor/TMEM97 is overexpressed in many tumours, and sigma‐2 receptor ligands are under investigation for cancer therapy. We intended to evaluate the effect of PB28 on renal cancer in proliferation, migration and invasion in vitro and in vivo. Invasive renal cancer cell lines treated with PB28 (or sigma‐2 receptor antagonist 1) were subjected to cell proliferation, migration and invasion assays. The therapeutic effect of PB28 was performed on nude mice. Western blot for proteins in the PI3K‐AKT‐mTOR signalling pathway was conducted. A CCK‐8 assay was used to examine the effect of the combination of PB28 and cisplatin on renal cancer cells. Significant inhibitory effects were observed on proliferation, migration and invasion of 786‐O and ACHN cells after culturing with PB28. But, the outcomes of sigma‐2 receptor antagonist 1 presented the opposite tendency. PB28 significantly inhibited the proliferative and invasive ability of OS‐RC‐2 cells in vivo. Treatment resulted in decreased phosphorylation of constituents of the PI3K‐AKT‐mTOR pathway. The combination of PB28 and cisplatin showed enhanced efficacy in the inhibition of renal cancer cell proliferation. Taken together, PB28 inhibited the tumorigenic behaviours of renal cancer cells by regulating the PI3K‐AKT‐mTOR signalling pathway and was expected to be a sensitizer of cisplatin.

## 1 INTRODUCTION

Renal cancer is a malignancy that originates in the renal tubular epithelial system and is one of the most common malignant tumours in the urinary system. Although surgical treatment is commonly performed, most patients still die of tumour recurrence and metastasis.^1^ The sigma receptor was first reported in 1976, and there are two identified subtypes: sigma‐1 and sigma‐2.^2^, ^3^ The sigma receptor has been of interest since its discovery due to its wide range of biological functions, and it is considered to play significant roles in regulating cell survival, morphology and differentiation.^4^, ^5^ It has gradually become a target for cancer diagnosis and treatment.^6^, ^7^, ^8^, ^9^ The sigma‐2 receptor is an 18–21 kD membrane protein,^2^ and recent receptor‐ligand binding assays have shown that it is highly expressed in the liver and kidney of rabbits.^10^ Overexpression of the sigma‐2 receptor was also observed in the central nervous system and proliferative tumours.^6^, ^11^, ^12^ At present, the sigma‐2 receptor has emerged as potential treatment target in malignant tumours and neurological diseases.^7^, ^13^, ^14^ Notwithstanding the increasing importance of the sigma‐2 receptor in the treatment of diseases, the process of further understanding its biological functions has been greatly hindered because genes encoding the sigma‐2 receptor have never been discovered. A breakthrough occurred in 2017, in which a study performed by Assaf Alo confirmed that TMEM97 encoded the sigma‐2 receptor.^15^ TMEM97 (also known as MAC30) is a member of the insulin‐like growth factor family and is relevant to many biological functions in the human body. These processes are related to liver development and differentiation, cholesterol metabolism and cancer transformation.^16^, ^17^, ^18^, ^19^ The high‐affinity sigma‐2 receptor ligand PB28 (1‐cyclohexyl‐4‐[3‐(5‐methoxy‐1,2,3,4‐tetrahydronaphthalen‐1‐yl)propyl]piperazine) has been recognized as a novel effective inhibitor of the proliferation of human breast cancer cell lines in vitro.^20^, ^21^ Furthermore, a recent study showed that PB28 could induce apoptosis by increasing the production of mitochondrial superoxide radicals in pancreatic cells and reducing tumour growth in vivo.^22^ In addition, PB28 was suggested to reverse the resistance of breast cancer cells to doxorubicin by downregulating P‐glycoprotein (P‐gp) expression levels.^20^ We suggest that the sigma‐2 agonist PB28 could be a novel anticancer drug either as a monotherapy or in a combination regimen. Herein, we assessed the efficacy of PB28 and sigma‐2 receptor antagonist 1 and a probable and unknown mechanism by which the sigma‐2 receptor/TMEM97 affects renal cancer. In our current study, we investigated the ability of PB28 to inhibit cell proliferation, migration and invasion by decreasing phosphorylation of constituents of the PI3K‐AKT‐mTOR pathway. Moreover, the enhanced chemosensitivity of PB28‐treated renal cancer cells to cisplatin was also studied.

## 2 MATERIALS AND METHODS

### 2.1 Cell lines and Compounds

Four renal cancer cell lines (786‐O, ACHN, Caki‐1 and OS‐RC‐2) and one human renal tubular epithelial cell line (HK‐2) were selected for culture (cells were all purchased from Chinese Academy of Sciences Cell Bank). The 786‐O and OS‐RC‐2 cells were maintained in RPMI 1640 (HyClone, Logan, USA) supplemented with 10% foetal bovine serum (FBS) (TBD, Tianjin, China) and cultured at 37°C in a 5% CO2 humidified incubator. ACHN, Caki‐1 and HK‐2 cells were cultured in MEM, McCoy's 5A and F12 (HyClone, Logan, USA) under the same conditions described above. PB28 (Santa Cruz Biotechnology, USA) is a highly selective agonist of sigma‐2, and sigma‐2 receptor antagonist 1 is a sigma‐2 receptor antagonist purchased from MedChemExpress (New Jersey, USA). Cisplatin was obtained from Sigma Inc.

### 2.2 Western Blot Analysis

Cells were washed with ice‐cold PBS (phosphate buffer saline) three times (performed on ice), lysed in RIPA buffer supplemented with PMSF (phenylmethanesulfonyl fluoride) protease inhibitor and centrifuged at 12,000 rpm for 30 min. Then, the BCA assay was used to determine the protein concentrations (Takara, Kyoto, Japan). Afterwards, 30 µg of standardized protein per lane was separated by SDS‐PAGE (sodium dodecyl sulphate polyacrylamide gel) through a 12% gel and transferred to PVDF (Polyvinylidene fluoride) membranes (Bio‐Rad, USA). The membranes were incubated overnight at 4°C with primary antibodies against β‐actin (1:5000) (Cell Signaling Technology, USA), PI3K‐110α (1:2000) (Cell Signaling Technology, USA), p‐AKT (1:1000) (Santa Cruz Biotechnology, USA), p‐mTOR (1:1000) (Cell Signaling Technology, USA), mTOR (1:2000) (Cell Signaling Technology, USA), AKT (1:1000) (Santa Cruz Biotechnology, USA), N‐cadherin (1:1000) (Abcam, Hong Kong) and Vimentin (1:1000) (Abcam, Hong Kong). All membranes were subsequently incubated with secondary anti‐mouse or anti‐rabbit IgG antibody (Zhongshanjinqiao, Beijing, China) at 37°C for 1 h on a shaking table, and bound proteins were detected using the EC3 Imaging System (UVP Inc., Cambridge, UK).

### 2.3 Proliferation Assay

Colony formation assay and RTCA (Real‐time cell analysis) assay were used to detect the proliferation of renal cancer cells. Colony formation assay: In the first group, cells (786‐O and ACHN) were seeded into 6‐cm cell culture dishes (1000–1500 cells per dish) and incubated with sigma‐2 receptor antagonist 1 (0 nM, 100 nM, 500 nM and 1000 nM) for 14 days. In the second group, 786‐O and ACHN cells were inoculated as mentioned above (1000–1500 cells per dish). Thereafter, cells were cultured with various concentrations of PB28 (0 μM, 1 μM, 5 μM and 10 μM). After 8–14 days, the proliferation of cells in both groups was determined by the crystal violet staining assay. RTCA assay: RTCA (ACEA Biosciences, USA) was performed to monitor cell viability according to the manufacturer's instructions. The xCELLigence software began to record the electrical values when cells adhere to the surface of the plate and influenced. The actual experiment began, cells were mixed with 100 μl of culture medium and seeded into plates after the background value measured. Culture medium containing PB28 (H₂O) or sigma‐2 receptor antagonist 1 (DMSO) was added, respectively. All data were documented with ACEA Biosciences RTCA software 2.0 and analysed by GraphPad Prism 5.

### 2.4 Invasion Assays

Transwell chambers were used to assess cell invasion. In all, 20 μl of Matrigel^®^ (BD Biosciences, Franklin Lakes, USA) was diluted fivefold with serum‐free medium and added to the upper chamber of a 24‐well transwell chamber. A total of 200 μl of cell suspension (diluted in RPMI 1640 or MEM with 1 – 2% FBS) at a density of 1 × 10^5^ cells were added to the upper chamber, while 600 μl of RPMI 1640 or MEM supplemented with 10% FBS was added to the lower chamber. Specific concentrations of PB28 (5 µM) and sigma‐2 receptor antagonist 1 (300 nM) were incubated for 24 h. Afterwards, cells that invaded through to the lower chamber were stained with crystal violet solution for ten minutes. Cells in five random fields were counted under a light microscope (Olympus, Tokyo, Japan).

### 2.5 Wound Healing Assay

Cells were seeded in a 24‐well plate and incubated at 37°C until they reached confluence. Then, the monolayer was scratched using a plastic tip and then washed two times with PBS to remove the cell fragments. Then, the cells were incubated with culture medium in the presence or absence of PB28 (10 µM) /sigma‐2 receptor antagonist 1 (300 nM). Microscopy images of the scratches were taken immediately after the scratch (zero hour) and at 24 h. Cell migration ability was assayed using ImageJ software.

### 2.6 In vivo assessment of tumour growth and lung metastasis

Animal experiments were performed according to the animal experiments protocol approved by the Ethics Committee of China Medical University. In this animal model, we utilized the OS‐RC‐2 cell line, which is aggressive with ideal implantation and tumour growth rate. In vivo experiments with nude mice were executed to compare the effect of PB28 with physiological saline solution. First, proliferation experiment group: female SPF/VAF nude mice (6 weeks old, Vitalriver China) were injected in the right flank with 150 μl (1 × 10^5^ cells per mouse) of a single‐cell suspension of OS‐RC‐2 cells in serum‐free RPMI 1640 and 50 μl matrigel (diluted with serum‐free RPMI 1640). Treatment began when the average tumour diameter was 3 mm. Second, invasion experiment group: mice were injected with 200 μl (4 × 10^4^ cells per mouse) of a single‐cell suspension of OS‐RC‐2 cells (diluted with PBS) into the caudal vein. Treatment began one week after the injection. Intervention methods in the experiment were as follows: mice treated with vehicle (the same volume of PB28) served as the control cohort. Mice received intraperitoneal (i.p.) injections of PB28 (0.6 mg) or control vector once every other day in 100 μl medium for (subcutaneous group two weeks; caudal vein group four weeks). Proliferation experiment group: tumours were measured once three days, and tumour volumes were calculated according to the formula = Length × Width^2^/2. The tumour specimens were harvested for immunohistochemical analysis (Ki‐67 and TMEM97 staining). Invasion experiment group: the lung specimens were harvested for haematoxylin‐eosin staining analysis to evaluate the number and volume of lung metastasis.

### 2.7 Immunohistochemistry staining and haematoxylin‐eosin (HE) staining

Immunohistochemistry staining: Fresh tissues harvested from nude mice were fixed with 4% formalin for 48 h. Sections were subsequently stained using Ki‐67 antibody (Cell Signaling Technology, USA) or TMEM97 antibody (Novus, USA). Then, all samples were stained with DAB (Zsjqbio, Beijing, China), and nucleus was stained with haematoxylin (Beyotime, Shanghai, China) for two minutes and rinsed with water for 20 min. For HE staining, lung tissues were paraffin‐embedded, dewaxed, rehydrated and stained with haematoxylin‐eosin. Finally, neutral balsam was used to seal the slides. Images achieved by Upright Metallurgical Microscope (Olympus, Tokyo, Japan) were used for analysis.

### 2.8 Cell Viability

Cell Counting Kit‐8 (CCK‐8) (Beyotime, Shanghai, China) assays were employed to determine the viability of ACHN and Caki‐1 cells incubated with cisplatin (included PB28). The first day, ACHN and Caki‐1 cells were plated at a density of 1.0 × 10^5^ cells/well in 96‐well plates 24 h prior to treatment. The next day, cells were treated with cisplatin (4–64 μg ml^−1^) in the presence or absence of PB28 (10–20 μM). After incubation for 24 h, CCK8/medium (1:100) was added to each well, and after a one‐hour incubation at 37°C, the OD values were obtained at 570 nm.

### 2.9 Statistical Methods

SPSS software for Windows 17.0 (SPSS Inc., Chicago, USA) was used to conduct statistical analyses. Student's t test was used to analyse differences in cell invasion and migratory. Analysis of variance of repeated measures was used to evaluate the RTCA results and the tumour growth curves in nude mice. One‐way ANOVA analysis was performed on the colony formation assay. P values < 0.05 were considered significantly.

## 3 RESULTS

### 3.1 PB28 Inhibited the Proliferation of Renal Cancer Cells while Sigma‐2 Receptor Antagonist 1 Induced the Proliferation of Renal Cancer Cells

**FIGURE 1:**
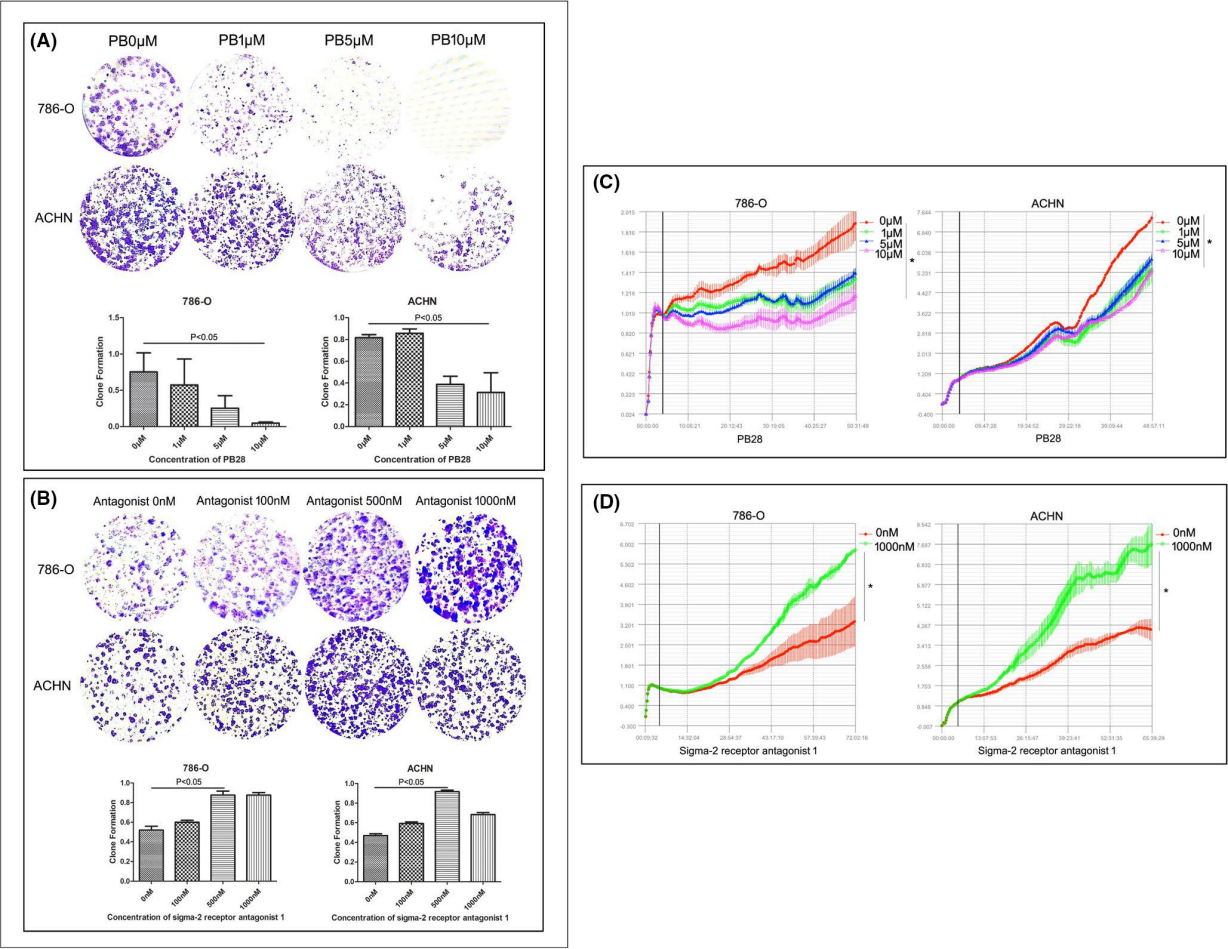
Effects of PB28 or sigma‐2 receptor antagonist 1on the proliferation of renal cancer cells. Colony‐forming assay and RTCA assay were used to evaluate the proliferative ability of renal cancer cells. A The clonogenicity of 786‐O and ACHN cells showed a significant decrease when the cells cultured with PB28 (786‐O: *p *= 0.038, ACHN: *p *= 0.031). C The RTCA analysis also demonstrated an inhibitory effect of PB28 on renal cancer cells. B The colony‐forming abilities of 786‐O and ACHN cells increased significantly after sigma‐2 receptor inhibition for 8–10 days (*p *= 0.0249 and *p *= 0.0153). D The RTCA analysis showed an facilitate effect of sigma‐2 receptor antagonist 1 on renal cancer cells. In the figure, PB represents PB28. YI represents sigma‐2 receptor antagonist 1. * represents *p *< 0.01

We detected the effect of PB28 on the proliferation of 786‐O and ACHN cells. As shown in Figure 1A, the proliferative ability of 786‐O and ACHN cells was measured by the colony‐forming assay, PB28 exerted a dose‐dependent inhibitory effect on the viability of both cancer cell lines. Neither of the two cell lines was resistant to PB28 (786‐O:*p *= 0.038, ACHN:*p *= 0.031). Sigma‐2 receptor antagonist 1 was applied to treat 786‐O and ACHN cells. Clonogenic assays were used to measure the impact of cell density on the colony‐forming ability of 786‐O and ACHN cells. After 8–10 days of incubation with sigma‐2 receptor antagonist 1, colony formation was significantly augmented (Figure 1B) (786‐O:*p *= 0.0249, ACHN:*p *= 0.0153). As for RCTA analysis, PB28 exhibited a dose‐dependent inhibitory effect on the viability of 786‐O and ACHN cells Figure 1C, *p* < 0.01). While 1000nM concentration of sigma‐2 receptor antagonist 1 increased the proliferative activity of both 786‐O and ACHN cells ( Figure 1D, *p* < 0.01).

### 3.2 PB28 Inhibited the Invasion and Migration of Renal Cancer Cells, while Sigma‐2 Receptor Antagonist 1 Promoted These Activities

**FIGURE 2:**
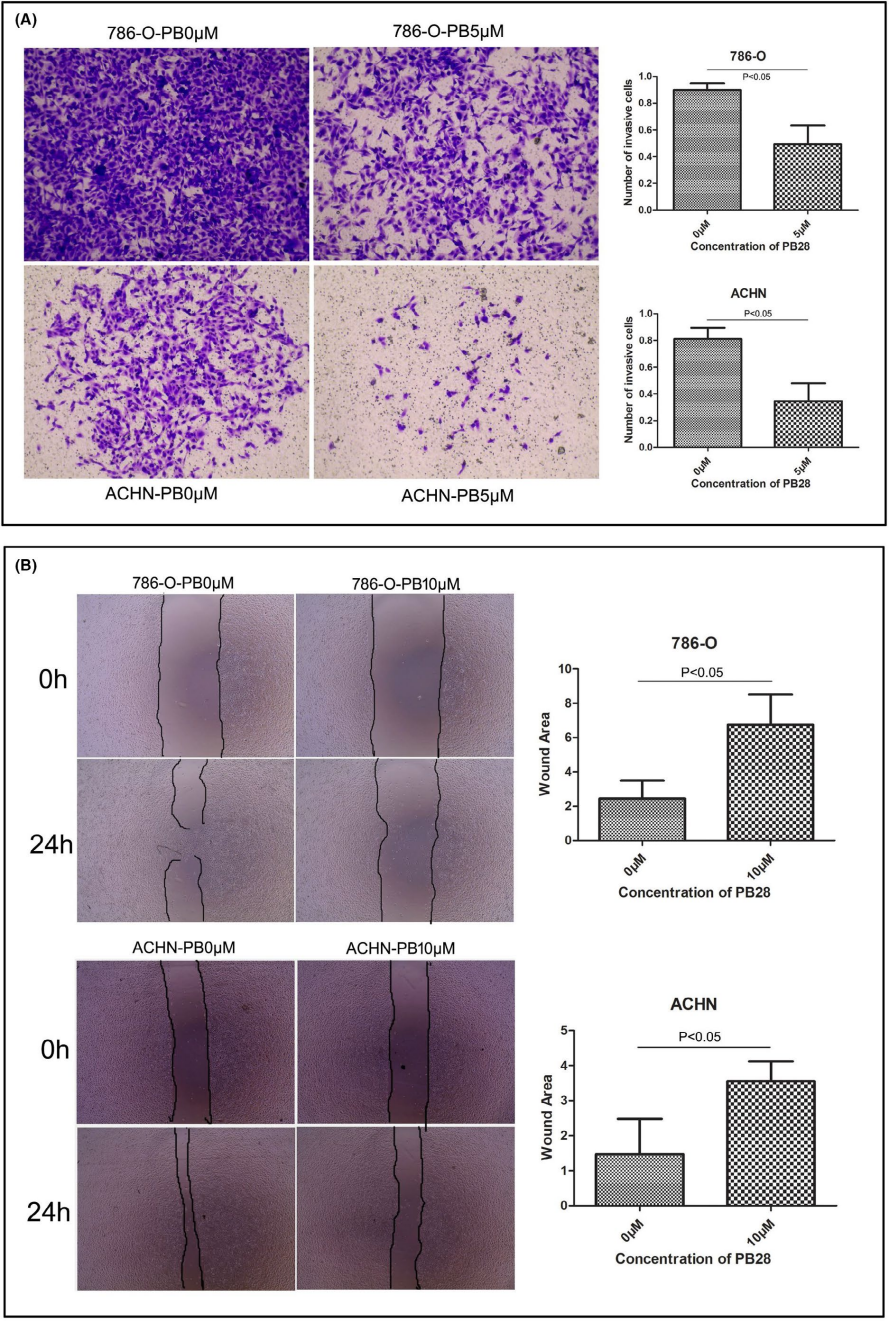
Effects of PB28 on the invasion or migration of renal cancer cells. Matrigel‐coated membranes were used to show the invasive ability of malignant renal cancer cells. A PB28 at 5 μM significantly reduced the invasion of both 786‐O and ACHN cells as revealed by the transwell assay (786‐O: *p *= 0.0356; ACHN: *p *= 0.024). B 786‐O and ACHN cells were seeded in 24‐well plates and scratched using a 1 ml pipette tip upon reaching confluence. Cell migration into the wound area was measured. Cells were treated with the sigma‐2 agonist PB28 and subjected to in vitro wound healing assays. Images were captured by an inverted light microscope. After comparing scratch widths to verify the migratory ability of cells treated with the compounds listed above at different concentrations, cells were counted after 24 h. PB28 obviously inhibited the migration of both 786‐O and ACHN cells at concentrations of 10 μM (P values were 786‐O: 0.0355, ACHN: 0.0477). In this figure, PB represents PB28

**FIGURE 3:**
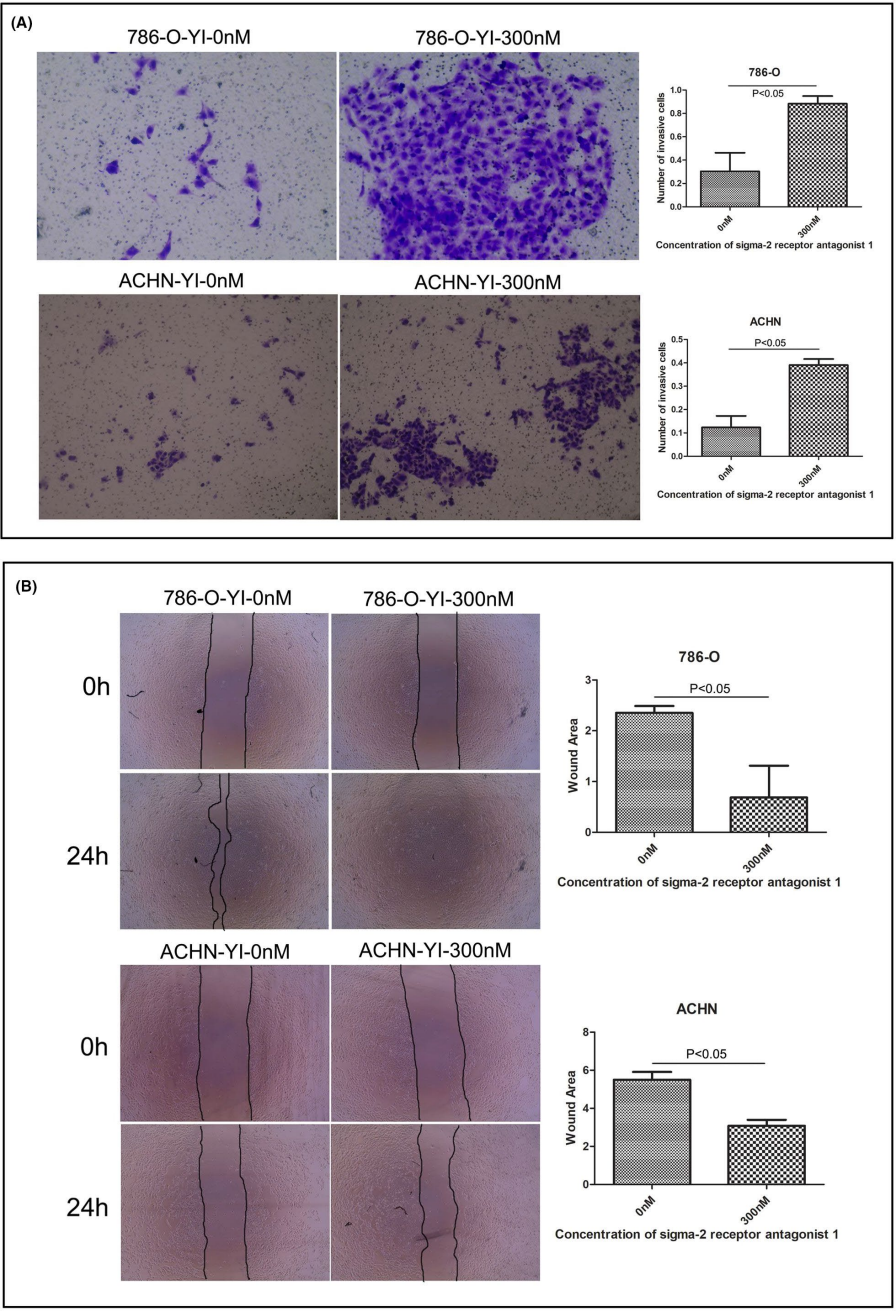
Effects of sigma‐2 receptor antagonist 1 on the invasion or migration of renal cancer cells. A Cells cultured with sigma‐2 receptor antagonist 1 (concentrations of 300 nM) greatly increased the invasive ability of 786‐O and ACHN cells, respectively. The P values were as follows: 786‐O, 0.0037 and ACHN, 0.0104. B Sigma‐2 receptor antagonist 1 exhibited a stimulative effect towards 786‐O and ACHN cells at concentrations of 300 nM. The P values were all significantly less than 0.05 (786‐O: 0.0322, ACHN: 0.0127). In the figure, YI represents sigma‐2 receptor antagonist 1

Tumour invasion is one of the main reasons for poor prognosis in patients with cancer. We investigated the potential role of PB28 and sigma‐2 receptor antagonist 1 in modulating the invasion and migration abilities of renal cancer cells by the transwell invasion assay with Matrigel and the wound healing assay, respectively. We observed that PB28 (5 μM) significantly inhibited the invasion of ACHN cells by more than 50%, and the invasion of 786‐O cells was also inhibited after PB28 (5 μM) treatment for 48 h (Figure 2A). Furthermore, as shown in Figure 2B, the in vitro migration assays showed that a low dose (10 μM) of PB28 significantly inhibited the migration of 786‐O cells and ACHN cells, respectively. Transwell invasion assays with Matrigel showed that the numbers of invading 786‐O and ACHN cells were increased after treatment with 300 nM sigma‐2 receptor antagonist 1 (Figure 3A). By contrast, sigma‐2 receptor antagonist 1 significantly accelerated the migration of 786‐O cells and ACHN cells at a low concentration (300 nM) (Figure 3B).

### 3.3 PB28 inhibited OS‐RC‐2 cell proliferation and invasion in vivo

**FIGURE 4:**
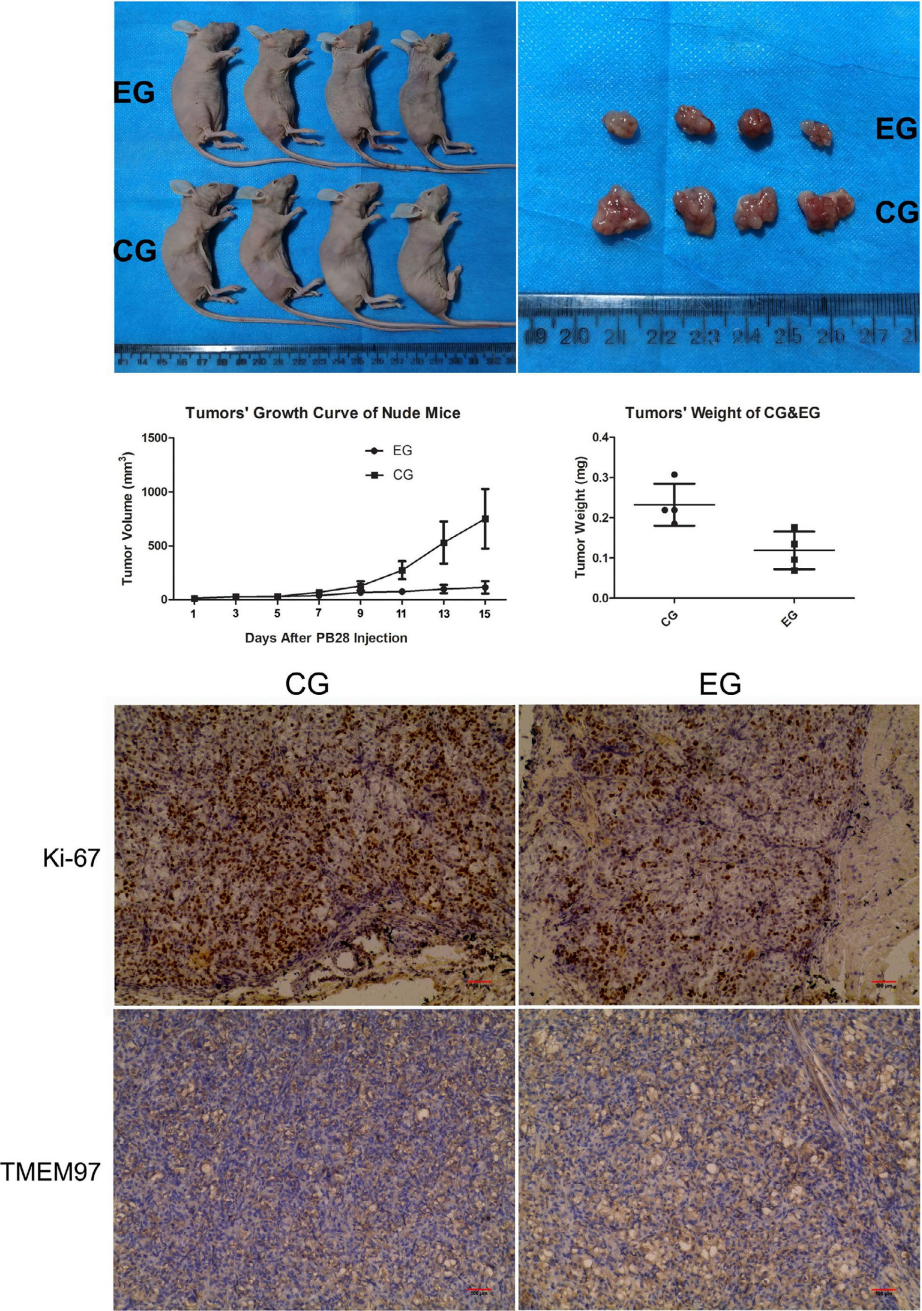
Representative images of tumours and tissue slices of nude mice. Tumours derived from EG (experimental group (i.p. with PB28)) grew at a slower rate and were smaller in size than tumours derived from CG (control group (i.p. with saline solution)). The volumes of the tumours on the flanks of the mice were recorded 1, 3, 5, 7 and 9 days after the injection of PB28. Tumours derived from control group grew at a faster rate (*p *< 0.0001) and were larger in volume (*p *= 0.00286) than tumours derived from experimental group on all days assessed. Ki‐67 (brown staining) localized to the nuclear in tumour tissues derived from OS‐RC‐2 cells. Furthermore, Ki‐67 presented a stronger staining in the control group compared with the experimental group and TMEM97 (brown staining) mainly localized to the cytoplasm in tumour tissues

**FIGURE 5:**
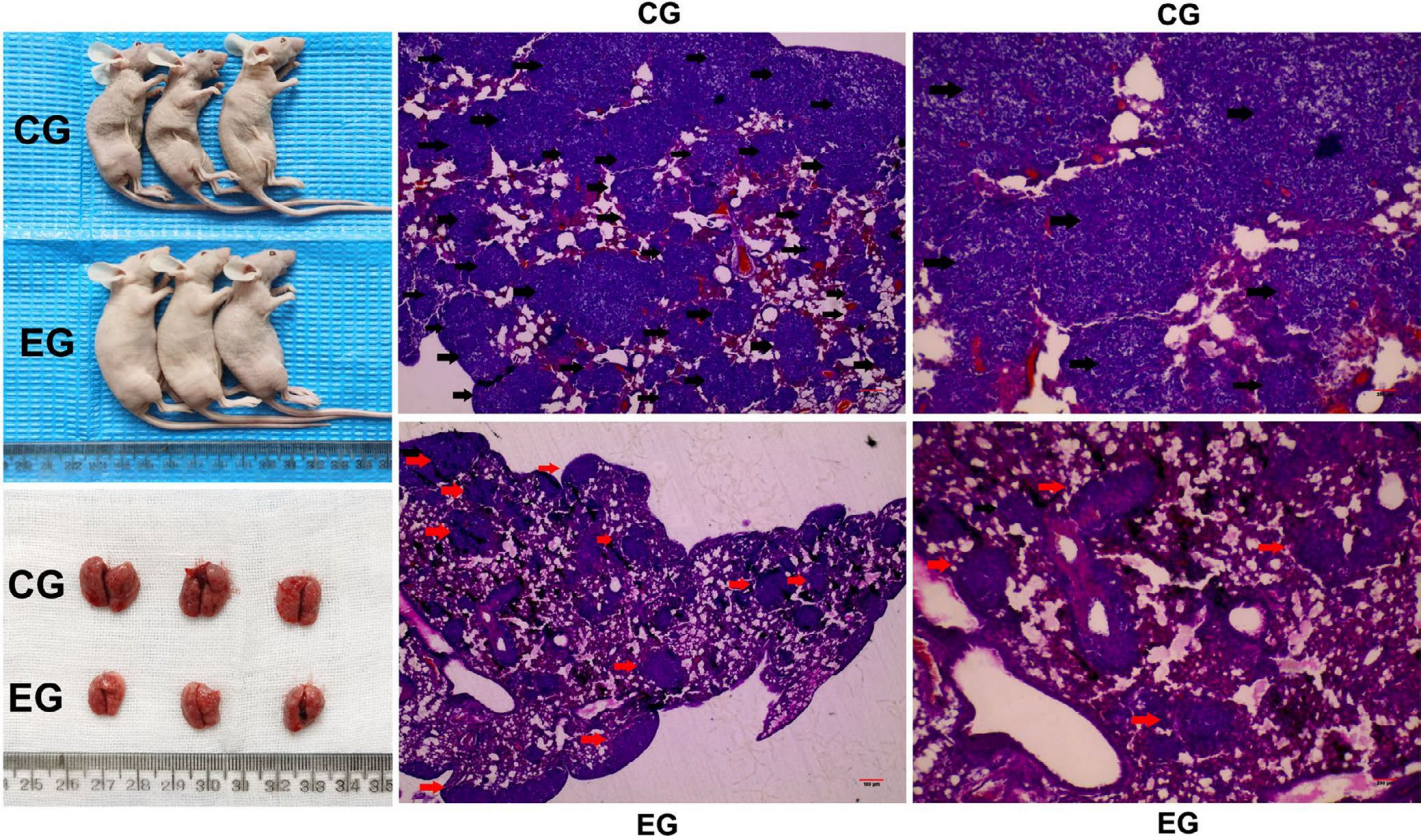
Representative images of nude mice, lung metastases and H&E staining slices. All mice were sacrificed followed a four‐week treatment of PB28. The number and volume of lung metastatic nodules (red arrows) in experimental group (EG) were significantly decreased compared to the control group (CG) (black arrows) (*p *< 0.0001)

We further investigated the effect of PB28 in vivo. OS‐RC‐2 cells were injected into the armpit (four mice per group) or the caudal vein (three mice per group) of nude mice. We found that tumours derived from PB28 treatment group grew at a slower rate (*p *< 0.0001) and were lighter than tumours derived from control group (*p *= 0.00286), and Ki‐67 presented a weaker staining (Figure 4). Furthermore, not surprisingly, images of gross specimens and HE staining, PB28 significantly reduced the number and volume of lung metastatic nodules (*p *< 0.0001) (Figure 5). The above results were consistent with the findings in vitro, implicating TMEM97 as a candidate tumour suppressor in renal cancer.

### 3.4 PB28 Suppressed the PI3K‐AKT‐mTOR Signalling Pathway

**FIGURE 6:**
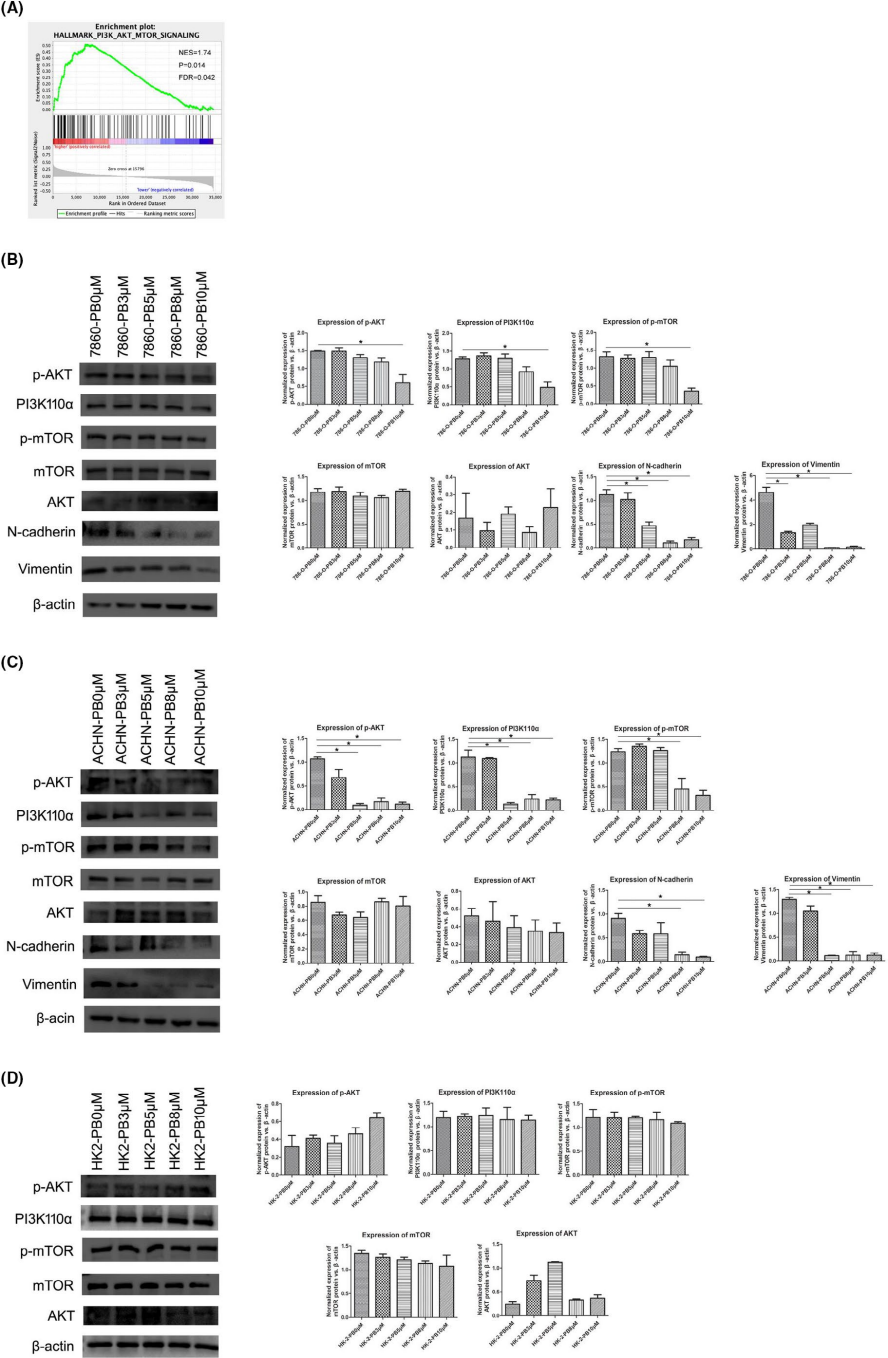
PB28 regulated the expression of proteins in the PI3K‐AKT‐mTOR signalling pathway and EMT. A Results from GSb EA bioinformatics analysis. B**‐**D Western blotting analysis of 786‐O, ACHN and HK‐2 cells treated with various concentrations PB28. The protein levels of PI3K‐110α, p‐mTOR and p‐AKT, mTOR, AKT, N‐cadherin and Vimentin were analysed, and β‐actin was measured to indicate protein loading. The ratios of target genes to β‐actin expression were determined in basal cells and were set at 1

According to the GESA bioinformatics results, we predicted that TMEM97 was positively associated with the PI3K‐AKT‐mTOR signalling pathway in renal cancer (Figure 6A). Then, Western blotting analysis was used to examine the expression of proteins related to this signalling pathway after PB28 treatment. The results showed that the expression of likely constituents in the PI3K‐AKT‐mTOR signalling pathway (PI3K‐110α, p‐mTOR and p‐AKT) was significantly inhibited by PB28 (AKT and mTOR were not influenced) in 786‐O and ACHN cells, and the expression of EMT‐related proteins (N‐cadherin and Vimentin) showed the same trend (Figure 6B, 6C). Nevertheless, PB28 had no effect on the PI3K‐AKT‐mTOR signalling pathway in HK‐2 cells (Figure 6D).

### 3.5 PB28 Increased the Sensitivity of Renal Cancer Cells to Cisplatin

**FIGURE 7:**
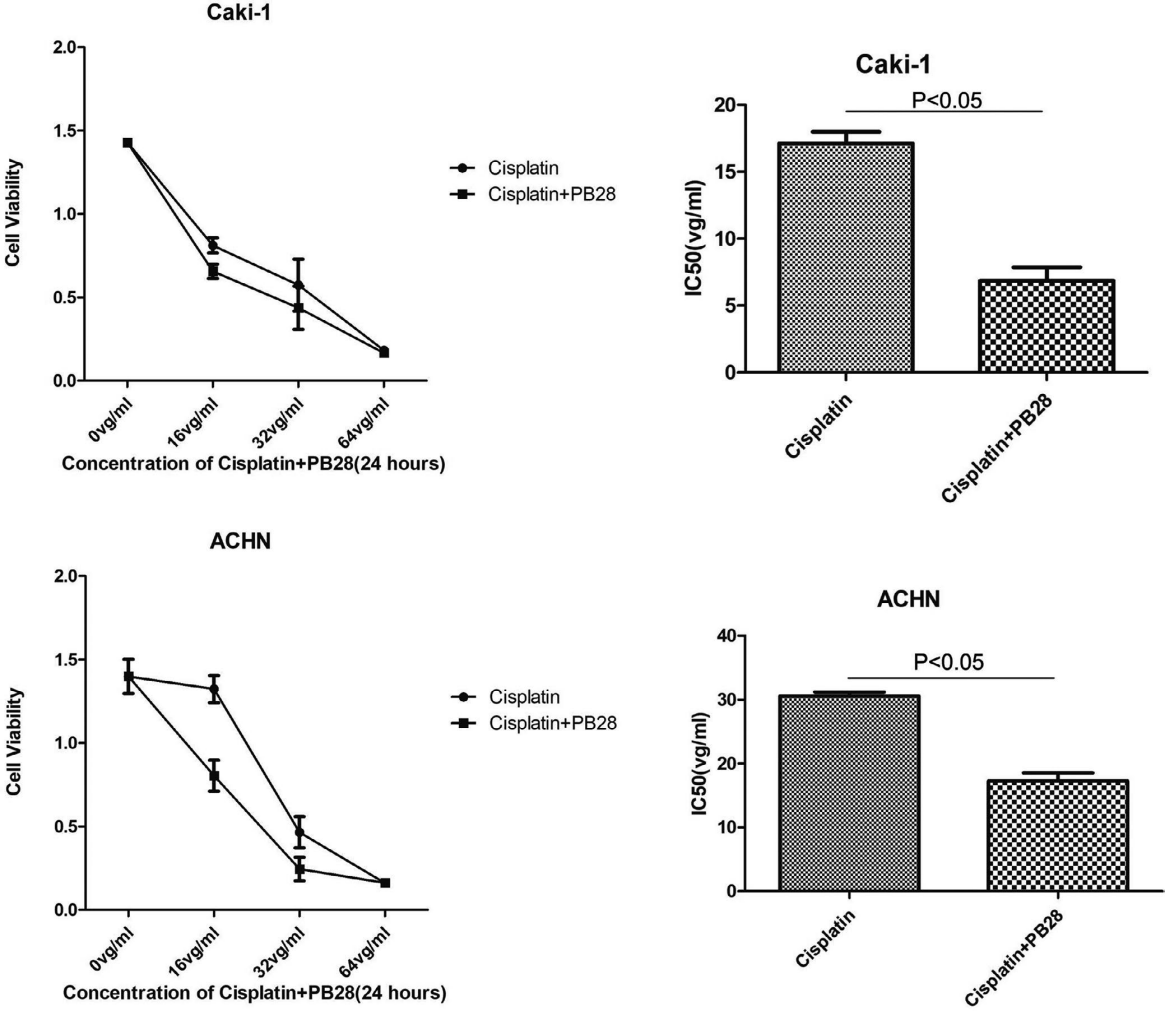
PB28 fortified the sensitivity of renal cancer cells to cisplatin. The CCK‐8 assay was performed to measure the change in the IC50 value of cisplatin in renal cancer cells with or without PB28. The IC50 of cisplatin in Caki‐1 cells decreased from 17.1 μg ml^−1^ to 6.86 μg ml^−1^ when PB28 was added (*p *= 0.0047) and that in ACHN cells decreased from 30.54 μg ml^−1^ to 17.28 μg ml^−1^ (*p *= 0.0039)

As multidrug resistance is common in human renal cancer, the effects of PB28 and cisplatin on renal cancer cells were investigated to study the potential implication of TMEM97 in drug sensitivity and in vitro chemoresistance. We examined three renal cancer cell lines, 786‐O, Caki‐1 and ACHN. The cells were incubated with cisplatin in the presence or absence of PB28 for 24 h. Cell viability was evaluated by a CCK‐8 assay, and the half‐maximal inhibitory concentration (IC50) of cisplatin (with or without PB28) was also examined. The IC50 values of cisplatin were as follows: 786‐O, 2.387 μg ml^−1^ (the result was not presented); Caki‐1, 17.1 μg ml^−1^; and ACHN, 30.54 μg ml^−1^. We speculated that 786‐O cells were more sensitive to cisplatin and that Caki‐1 and ACHN cells were resistant to cisplatin. Therefore, we selected Caki‐1 and ACHN cells for the following experiment. We added a low concentration of PB28 (Caki‐1: 10 μM, ACHN: 20 μM) to the medium of the two cell lines. After a 24 h incubation, the CCK‐8 results showed a sharp drop in the IC50 value of cisplatin in Caki‐1 (6.86 μg ml^−1^) and ACHN (17.28 μg ml^−1^) cells (Figure 7). We hypothesized that PB28 could enhance cisplatin efficacy in renal cancer cells with few side effects.

## 4 DISCUSSION

Renal cancer is a malignancy that increases the burden of human cancers and has a poor prognosis rate, and there are difficulties in the clinical management of distant metastasis.^23^ Sigma receptors and their ligands function in processes beyond those related to cell differentiation and survival, including cholesterol metabolism, cancer cell growth and invasion.^17^, ^18^, ^19^ Furthermore, TMEM97 was identified as a sigma‐2 receptor ligand in 2017, which expanded the study of sigma receptor function.^15^ It was reported that TMEM97 has been confirmed to be a factor associated with cancer progression,^18^, ^19^, ^23^ but the related regulatory signalling pathways were not further studied. To this end, we investigated the mechanism of TMEM97 in renal cancer.

First, the sigma‐2 receptor has been reported to be expressed in different types of tumours, including gastric, pancreatic and breast tumours.^18^, ^19^, ^20^ In parallel, exogenous sigma‐2 receptor ligands have been confirmed as antitumour therapies.^7^, ^8^, ^9^, ^24^ Among many sigma‐2 receptor ligands, PB28 (as well as agonist) has exhibited exciting outcomes in breast and pancreatic cancer therapy.^20^, ^22^ Based on our experimental results, 786‐O and ACHN cells cultured with PB28 exhibited a reduction in proliferation, migration and invasion compared with those in control cells. Therefore, we hypothesized that TMEM97 plays a vital role in the development of renal cancer, PB28 might serve as a new therapeutical agent for renal cancer treatment. Then, sigma‐2 receptor antagonist 1 was added to cultured renal cancer cells. According to the matrigel and migration experiments, the results indicated that sigma‐2 receptor antagonist 1 promoted the invasion and migration of both 786‐O and ACHN cells. Colony formation assay and RTCA assay were also selected to determine the effect of sigma‐2 receptor antagonist 1 on renal cancer cells. Then, encouraging results were observed: the proliferative ability of 786‐O and ACHN cells increased significantly with sigma‐2 receptor antagonist 1 treatment. Therefore, the proliferative, migratory and invasive abilities of renal cancer cells showed an increasing trend when sigma‐2 receptor antagonist 1 was added to the cells. According to the results of above experiments, agonist and antagonist of sigma‐2 receptor play diverse roles in renal cancer. We speculated that TMEM97 might act as a tumour suppressor during the development of renal cancer, and PB28 had a therapeutic effect on it.

Second, we had confirmed an effective treatment of PB28 in vitro on renal cancer cells. Meanwhile, a nude mice experiment also came to a similar conclusion. Both the volume of subcutaneous tumours and the number (scope) of lung metastasis were significantly decreased by PB28 in vivo. Taken together, in vitro and in vivo experiment results suggested a latent vital role of PB28 in renal cancer therapy. However, the mechanism was unknown. A study by Pati ML, et al. suggests that the mitochondrial superoxide pathway is activated by PB28 in the treatment of pancreatic tumours.^22^ Furthermore, PB28 induces P‐gp down‐modulation through the activation of the caspases enrolled in both extrinsic and intrinsic apoptotic pathways. It can be used for treatment of multiple myeloma.^25^ Therefore, PB28 acted as an anticancer agent could induce cell death by different mechanisms. How about renal cancer?

Third, we predicted probable signalling pathway correlations with TMEM97 in renal cancer by studying bioinformatics analysis. The GSEA results suggested that TMEM97 is positively correlated with the PI3K‐AKT‐mTOR signalling pathway in renal cancer. Coutte L et al. demonstrated that the PI3K/AKT pathway is highly activated in renal cell cancer.^26^ Therefore, we evaluated the expression of proteins involved in the PI3K‐AKT‐mTOR signalling pathway and EMT in cells treated with PB28. We found that PI3K‐110α, p‐mTOR and p‐AKT, N‐cadherin, and Vimentin levels were decreased after PB28 was added, indicating that PB28 binding to its receptor TMEM97 could inhibit the subunit of PI3K, phosphorylation of AKT and mTOR (AKT and mTOR were not influenced) to suppress renal cancer cell growth and metastasis. However, the changes mentioned above had not happened in HK‐2 cells. We suspected that TMEM97 take effect on pathological or physiological status of renal via different signalling pathways.

Finally, because renal tumours are not very sensitive to chemotherapeutic drugs and the incidence of multidrug resistance has skyrocketed in recent years,^27^, ^28^ new chemical agents and combined chemotherapy regimens are urgently needed. Sigma‐2 agonists could modulate the expression of MDR‐1 and reduce the expression of P‐gp in numerous types of tumour cells, which provided the first evidence of this type of drug reversal of multidrug resistance in 1997.^29^ Furthermore, Francesco Berardi et al. demonstrated that PB28 reversed doxorubicin chemoresistance in breast cancer cells in a P‐gp‐dependent manner.^21^ The above results indicated that sigma‐2 agonists play a significant role in the resensitization of chemoresistant tumours. Hence, we chose three renal cancer cell lines, 786‐O, Caki‐1 and ACHN, to examine the IC50 value of cisplatin. As expected, we observed that Caki‐1 and ACHN cells were resistant to cisplatin, with IC50 values of 17.1 μg ml^−1^ and 30.54 μg ml^−1^, respectively. Combined treatment comprising cisplatin and PB28 resulted in a significant decrease in the IC50 value stated above. Our findings were consistent with the hypothesis that sigma‐2 agonists can enhance chemotherapy efficacy in renal cancer cells and identified PB28 as not only a therapeutic agent but also a novel sensitizer of cisplatin in renal cancer.

## 5 CONCLUSION

In conclusion, PB28 binding to TMEM97 might inhibit phosphorylation of constituents of the PI3K‐AKT‐mTOR signalling pathway, resulting in decreased proliferation, migration and invasion of renal cancer in vitro and in vivo. In parallel, PB28 increased the sensitivity of renal cancer cell lines to cisplatin.

## CONFLICT OF INTEREST

The authors declare that they have no conflict of interest.

## AUTHOR CONTRIBUTION

**Bo Zhan:** Data curation (lead); Formal analysis (lead); Writing‐original draft (lead); Writing‐review & editing (lead). **Zhe Zhang:** Investigation (equal); Methodology (equal); Project administration (equal); Resources (equal); Software (equal); Validation (equal); Visualization (equal). **Chiyuan Piao:** Investigation (equal); Methodology (equal); Project administration (equal); Resources (equal); Software (equal); Validation (equal); Visualization (equal). **Xiao Dong:** Funding acquisition (equal); Investigation (equal); Methodology (equal); Project administration (equal); Resources (equal); Software (equal); Validation (equal); Visualization (equal). **Yang Du:** Investigation (equal); Methodology (equal); Project administration (equal); Resources (equal); Software (equal); Validation (equal); Visualization (equal). **Chuize Kong:** Conceptualization (equal); Funding acquisition (lead); Project administration (lead); Resources (lead); Supervision (lead). **Yuanjun Jiang:** Conceptualization (equal); Project administration (equal); Supervision (equal).

## Data Availability

The data sets can be provided by corresponding author upon reasonable requests. *Chuize Kong* https://orcid.org/0000–0002–0875–2173
